# Integrative analysis of synovial sarcoma transcriptome reveals different types of transcriptomic changes

**DOI:** 10.3389/fgene.2022.925564

**Published:** 2022-09-02

**Authors:** Zhengwang Sun, Mengchen Yin, Yi Ding, Zixu Zhu, Yangbai Sun, Kun Li, Wangjun Yan

**Affiliations:** ^1^ Department of Musculoskeletal Oncology, Fudan University Shanghai Cancer Center, Shanghai, China; ^2^ Longhua Hospital, Shanghai University of Traditional Chinese Medicine, Shanghai, China; ^3^ Shanghai Key Laboratory of Regulatory Biology and School of Life Sciences, East China Normal University, Shanghai, China; ^4^ No.2 High School of East China Normal University, Shanghai,200000, China

**Keywords:** synovial sarcoma, gene expression, alternative splicing, gene fusion, circular RNA

## Abstract

**Background:** Synovial sarcoma (SS) is a rare and aggressive cancer that can come from distinct soft tissue types including muscle and ligaments. However, the transcriptomic landscape of SS is still poorly understood. This study aimed to systematically dissect the changes in SS transcriptome from different perspectives.

**Methods:** We performed deep total RNA sequencing on ten paired Synovial sarcoma and tumor-adjacent tissues to systematically dissect the transcriptomic profile of SS in terms of gene expression, alternative splicing, gene fusion, and circular RNAs.

**Results:** A total of 2,309 upregulated and 1,977 downregulated genes were identified between SS and tumor-adjacent tissues. Those upregulated genes could lead to the upregulation of the cell cycle, ribosome, and DNA replication pathways, while the downregulated genes may result in the downregulation of a set of metabolic biological processes and signaling pathways. Moreover, 2,511 genes (including 21 splicing factors) were differentially alternative spliced, indicating that the deregulation of alternative splicing could be one important factor that contributes to tumorigenesis. Additionally, we identified the known gene fusions of SS18-SSX1/SSX2 as well as 11 potentially novel gene fusions. Interestingly, 49 circular RNAs were differentially expressed and their parental genes could function in muscle contraction and muscle system processes.

**Conclusions:** Collectively, our comprehensive dissection of the transcriptomic changes of SS from both transcriptional and post-transcriptional levels provides novel insights into the biology and underlying molecular mechanism of SS.

## Introduction

Synovial sarcoma (SS) is a rare and aggressive soft tissue cancer, which tends to occur near large joints, particularly in the extremities of the arms or legs, in young adults (Ladanyi et al., 2002). At present, surgery is still the main treatment strategy for SS. Cytogenetically, a significant portion of SS cases involve nonrandom translocations between SS18 and SSX (Przybyl et al., 2012). Although a range of studies has investigated the genetic profile of SS from different cascades, a comprehensive transcriptomic profile of SS from different aspects is still lacking (Cancer Genome Atlas Research Network and Electronic address, 2017; He et al., 2017). RNA sequencing (RNA-Seq) technologies provide unprecedented opportunities to gain insights into the transcriptome from various aspects, including expression level, alternative splicing (AS), gene fusions, and circular RNAs. These analyses are essential to systematically reveal and better understand the abnormally transcriptomic changes of SS; however, a comprehensive exploration of the SS transcriptome from these aspects is still currently lacking.

AS is a crucial mechanism of post-transcriptional modification responsible for increasing both transcriptome and proteome diversity of a cell in eukaryotes (Wang et al., 2008; Keren et al., 2010). Since AS play important role in a variety of physiological processes (e.g. developmental programming), the misregulation of AS can result in splicing defects which may have a pathogenic function to cause severe diseases, including cancers (Wang and Cooper, 2007; Zhang and Manley, 2013). However, the genome-wide AS profile of SS is rarely studied to date. Furthermore, besides the common gene fusions formed by the translocation between chromosome X and 18 in SS, other gene fusions could also contribute to tumorigenesis or progression (Edwards, 2010). In addition, many circular RNAs (circRNAs) have been demonstrated to be functional as miRNA sponges and modulators of transcription (Chen, 2016; Li et al., 2018), which could be vital for different aspects of malignant phenotypes, such as cell cycle, apoptosis, and invasion (Qu et al., 2015; Greene et al., 2017). Moreover, some circRNAs are potentially important biomarkers for certain cancers (Abu and Jamal, 2016; Dong et al., 2017; Greene et al., 2017). But little is known about the expression profile of circRNAs in SS and almost no study has investigated this in SS. Thus, systematic dissection of the SS transcriptome from both transcriptional and post-transcriptional layers is necessary to better understand the underlying mechanisms of SS development.

Here we performed Ribo-Zero RNA-seq on ten pairs of Chinese SS and corresponding tumor-adjacent tissues to comprehensively explore the transcriptome profile of SS from various aspects. We first carried out differential expression calling and detected a number of upregulated and downregulated genes. Then the AS deregulation of a multitude of genes and a set of tumor-specific gene fusion events were identified. We also investigated the expression changes of circRNAs between SS and tumor-adjacent tissues. Moreover, we constructed an interaction network among circRNAs, miRNAs, and their target genes, which enabled us to further gain insights into the potential function of circRNAs in SS.

## Materials and methods

### RNA extraction

Total RNA was extracted from the 10 mg synovial sarcoma and tumor-adjacent tissues after grinding by Homogenizer (Scientz) using TRIzol^®^ Reagent (Invitrogen) and RNeasy MinElute spin column (Qiagen) according to the manufacturer’s instructions. Then the integrity of the total RNA was determined by 2100 Bioanalyser (Agilent) and quantified using the NanoDrop (Thermo Scientific). About 1 ug high-quality or media-quality RNA sample (OD260/280 = 1.9–2.0, RIN≥4) was used to construct the sequencing library.

### Library preparation and RNA sequencing

RNA purification, reverse transcription, library construction, and sequencing were performed at WuXi NextCODE in Shanghai according to the manufacturer’s instructions (Illumina). The rRNA-depleted sequencing libraries from total RNA were prepared using Illumina TruSeq^®^ Stranded Total RNA Gold preparation Kit. About 1 ug total RNA was used as input material, and then the Ribo-Zero Gold kit was used to remove both cytoplasmic and mitochondrial rRNA. After purification of the remaining RNA without rRNA, the RNA was fragmented into small pieces using divalent cations under elevated temperature. The cleaved RNA fragments are copied into the first-strand cDNA using reverse transcriptase and random primers, followed by second-strand cDNA synthesis. These cDNA fragments then were subjected to end-repair, phosphorylation, and ‘A’ base addition according to Illumina’s library construction protocol. The products were purified and enriched with PCR, and the AMPure XP Beads (Beckmen) were used to clean up the amplified target fragments to create the final cDNA library. After library construction, Qubit 2.0 fluorometer dsDNA HS Assay (Thermo Fisher Scientific) was used to quantify the concentration of the resulting sequencing libraries, while the size distribution was analyzed using Agilent BioAnalyzer 2100 (Agilent). Sequencing was performed using the Illumina system following Illumina-provided protocols for 2 x150 paired-end sequencing in WuXi NextCODE at Shanghai, China.

### Short-read mapping and gene expression quantification

The RNA-seq reads of each sample for 10 pairs of SS and tumor-adjacent tissues were separately aligned to the human reference genome GRCh38 using HISAT2 (version 2.1.0) (Kim et al., 2015). Then we quantified the gene expression of each sample by employing StringTie (version 1.3.6) (Pertea et al., 2015). The human gene annotation file in the GTF format of version 95 from the Ensembl database (http://www.ensembl.org) was used. The mapped read count and expression value in transcript per million (TPM) for each gene were obtained from StringTie and used for downstream analysis.

### Differential gene expression calling

For differential expression analysis, the read count mapped to each gene was used as input. The gene expression changes between SS and tumor-adjacent tissues were examined using DESeq2 (version 1.24.0) (Love et al., 2014). We defined the differentially expressed genes (DEGs) with the threshold of |fold change| >2 and adjusted *p*-value < 0.01.

### Detection of alternative splicing events

We investigated the alternative splicing (AS) profile of genes between SS and tumor-adjacent tissues by employing rMATS (version 4.0.2) (Shen et al., 2014). The bam files outputted by HISAT2 after read mapping were used as the input. Five common AS types of exon skipping (ES), alternative 3′ acceptor sites (A3AS), alternative 5′ donor sites (A5DS), intron retention (IE), and mutually exclusive exons (ME) were investigated. The differential alternative splicing events were identified with the cutoff of FDR <0.05.

### Identification of gene fusions

In order to explore the genetic alterations, we employed TopHat-Fusion (version 2.1.0) with default parameters to identify the gene fusion events in all tumor and normal samples (Kim and Salzberg, 2011). Only the fusions with at least 3 supporting reads and 2 supporting pairs were considered. Finally, 14 and 11 gene fusion pairs were detected in SS and tumor-adjacent tissues, respectively. We only kept the 14 gene fusions that are unique to SS and discarded the fusions detected in tumor-adjacent tissues.

### Circular RNA detection and differential expression analysis

We investigated the expression profiles of circRNAs in SS and tumor-adjacent tissues using CIRI (version 0.1.0) (Gao et al., 2015). Then differential expression analysis was conducted by employing DESeq2 (version 1.24.0) based on the expression count of circRNAs identified by CIRI. Only the circRNAs with expression changes of |fold change| >2 and adjusted *p*-value < 0.01 were considered as differentially expressed. The official IDs of circRNAs were obtained by coordinate mapping using the circBase database (Glazar et al., 2014).

### Construction of interaction network among circRNAs, miRNAs, and target genes

To gain insights into the function of circRNAs, we built an interaction network among the circRNAs, miRNAs, and the target genes of miRNAs. The PPI interactions were downloaded from the STRING database (version 11.0) (Szklarczyk et al., 2019). The regulatory relationship between miRNAs and target genes, as well as the known miRNA-circRNAs interactions, were obtained from the starBase database (version 3.0) (Li et al., 2014). We only used the circRNA-miRNA pairs supported by > 5 CLIP-seq experiments and the miRNA-target gene pairs supported by > 2 CLIP-seq experiments and >2 degradome-seq experiments in the StarBase2 database. Then we incorporated these interactions to construct the interaction network among circRNAs, miRNAs, and the genes targeted by miRNAs using Cytoscape (version 3.7.2) (Shannon et al., 2003). Only the parental genes of differentially expressed circRNAs, DEGs, DASGs, and fusion genes were considered in the interaction network construction.

### Gene functional enrichment analysis

We conducted gene ontology (GO) and KEGG pathway enrichment analyses using GSEA (version 4.0.1) for the upregulated and downregulated DEGs between SS and tumor-adjacent tissues (Subramanian et al., 2005). The functional enrichment analysis of biological processes and pathways for the differentially alternative spliced genes, fusion genes, and the parental genes of circular RNAs were carried out with cluster Profiler (version 3.12.0) (Yu et al., 2012). The enriched items with adjusted *p*-value < 0.05 were defined as significant.

## Results

### An abundance of important genes is differentially expressed between SS and tumor-adjacent tissues

**FIGURE 1:**
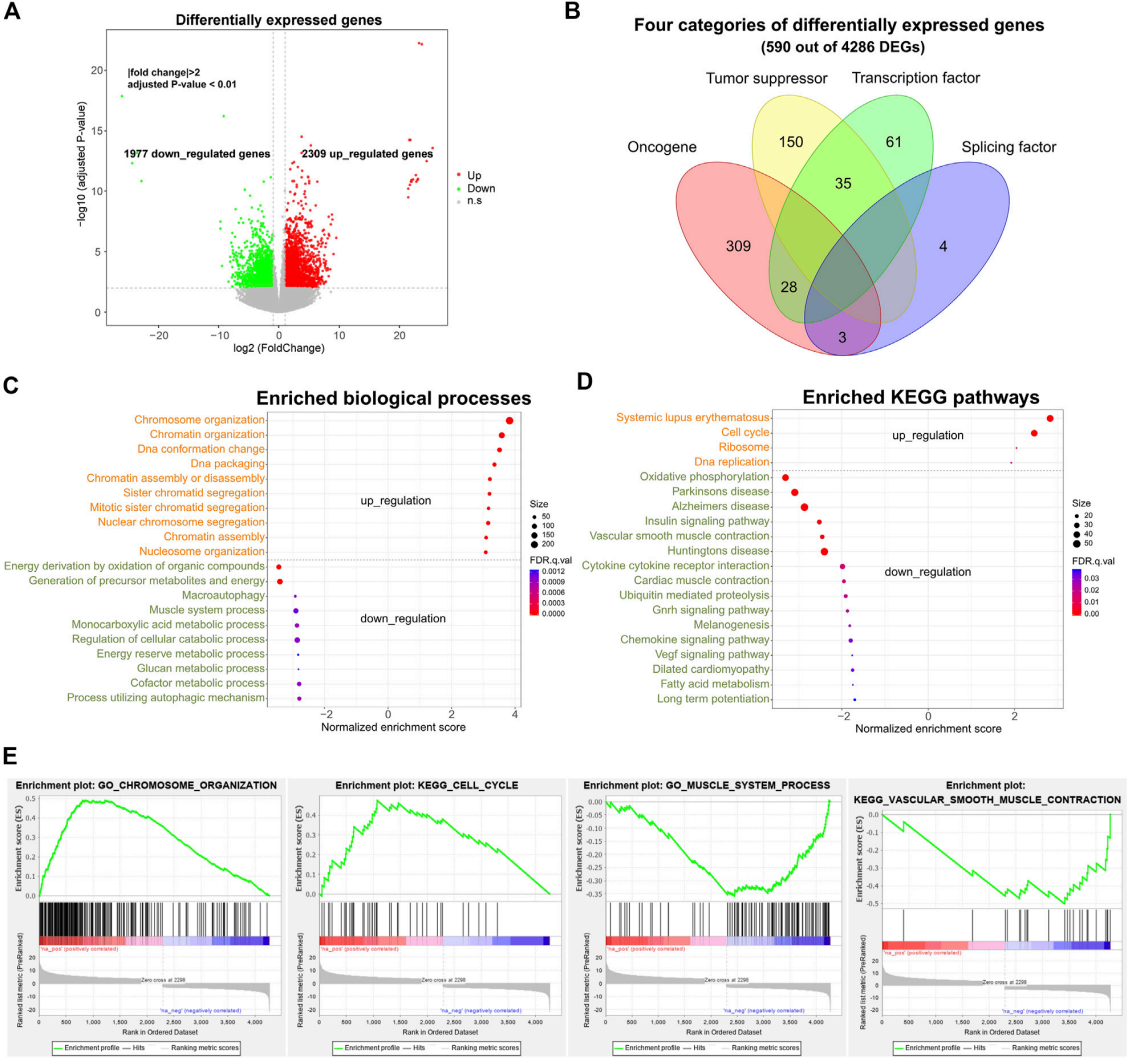
Differential expression profile and corresponding gene functions of SS. **(A)** Volcano plot displaying differentially expressed genes (DEGs) between ten pairs of SS and tumor-adjacent tissues. **|**fold change**|** >2 and adjusted *p*-value < 0.01. **(B)** Different categories of DEGs. **(C)** Top enriched up-regulated and down-regulated biological processes of DEGs. **(D)** Top enriched up-regulated and down-regulated KEGG pathways for DEGs. **(E)** Examples of enriched biological processes and pathways. Adjusted *p*-value < 0.05. DEGs: differentially expressed genes.

To gain insights into the transcriptomic changes of SS patients, we deeply sequenced the tumor and tumor-adjacent tissues of 10 SS patients with total RNA sequencing (including both poly (A+) and poly (A-) RNAs) (Table 1). We first aligned the RNA-seq reads of each sample to the human reference genome GRCh38 using HISAT2 (Kim et al., 2015) and then conducted differential expression calling by employing DEseq2 (Love et al., 2014). A total of 4,286 differentially expressed genes (DEGs) were detected using the threshold of |fold change| >2 and adjusted *p*-value < 0.01, of which 2,309 (including 432 lncRNA genes) and 1,977 (including 333 lncRNA genes) genes were separately upregulated and downregulated in SS compared to tumor-adjacent tissues (Figure 1A, Supplementary Table S1). Interestingly, we found that 340, 185, 124, and 7 of those DEGs are oncogenes, tumor suppressor genes (TSGs), transcription factors (TFs), and splicing factors (see Supplementary Figure S1 for differentially expressed splicing factors), respectively (Figure 1B). Specifically, 52 TFs (such as AES and BCL6) were down-regulated and 72 TFs (e.g. ARID3A and BRCA2) were up-regulated, suggesting that the expression changes of these TFs could influence the expression of their downstream target genes including related oncogenes and TSGs. Since oncogenes and TSGs are closely correlated with cancer, their expression changes may play an important role in the development of SS. Specifically, in consideration of the crucial function of splicing factors in AS regulation (Lee and Rio, 2015), we further conducted a qPCR experiment to validate the expression profiles of the seven splicing factors (ELAVL2, HNRNPA1, HNRNPH2, MBNL1, PCBP1, QKI, and TIA1) in DEGs (Supplementary Figure S2). As expected, the experimental results were consistent with the RNA-seq data, indicating the robustness of our analysis. Therefore, the differential expression of these splicing factors could result in the AS deregulation of corresponding genes in SS.

**TABLE 1:** Detailed information of 10 synovial sarcoma patients.

Patient ID	Age	Sex	Tumor Location	Tumor Size (cm)	Tumor status	Outcome
1	26	Female	Thigh	12*10.5*10	Primary	Alive
2	18	Male	Foot	6.0*3.5*2.0	Local recurrence	Died
3	37	Male	Groin	9.5*6*6	Local recurrence	Alive
4	29	Female	Lung	2.0*2.0*1.3	Primary	Alive
5	59	Female	Iliac Bone	5.5*4.5*3.5	Local recurrence	Alive
6	28	Female	Foot	2*1.7*0.7	Primary	Alive
7	20	Female	Neck	6*5*4	Primary	Alive
8	27	Male	Shank	7*6*2	Primary	Alive
9	41	Male	Shank	8*6*4	Local recurrence	Alive
10	71	Female	Thigh	9*6*3	Primary	Alive

Gene ontology (GO) and KEGG pathway enrichment analyses showed that these upregulated and downregulated DEGs were mainly involved in the fundamental and tumor-related biological processes and pathways (Figures 1C–E FDR <0.05). For example, the up-regulated DEGs were primarily enriched in the cell-cycle-related biological processes (e.g. chromosome organization, chromatin organization, and DNA conformation change) and pathways of systemic lupus erythematosus, cell cycle, DNA replication, and P53 signaling (Figure 1C). Several previous studies also identified the cell-cycle-related genes in sarcoma as a major category of up-regulated genes (Chibon et al., 2010; Yen et al., 2012), which was in line with our findings. By contrast, the down-regulated DEGs were mainly involved in the metabolic-related biological processes (such as energy derivation by oxidation of organic compounds, muscle system process, and glucan metabolic process) and the pathways of oxidative phosphorylation, insulin signaling pathway, and vascular smooth muscle contraction (Figure 1D). Thus, the result suggests that a multitude of genes prominently altered their expression levels in SS, which could be one of the main factors accounting for tumorigenesis through up-regulating and down-regulating corresponding pathways.

### Deregulation of alternative splicing could contribute to the tumorigenesis of SS

**FIGURE 2:**
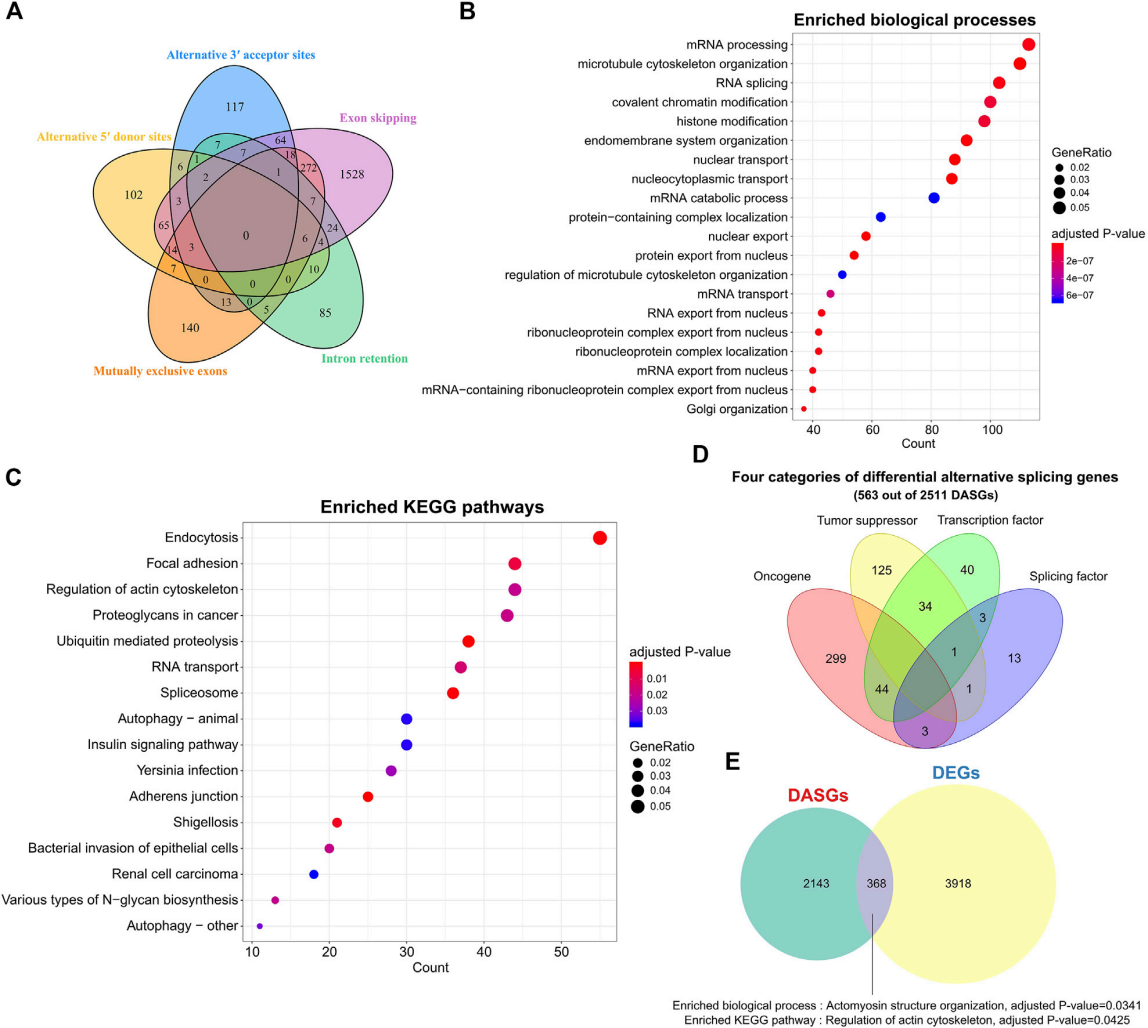
Alternative splicing patterns and related gene functions of SS. **(A)** Venn plot of the DASGs for five common splicing modes. **(B)** Top 20 enriched biological processes of DASGs. FDR <0.05. **(C)** Top 20 enriched pathways for DASGs. Adjusted *p*-value < 0.05. **(D)** Distinct categories of DASGs. **(E)** Comparison between DEGs and DASGs. DASGs: differentially alternative spliced genes; DEGs: differentially expressed genes.

Considering that the misregulation of AS can lead to the production of aberrant proteins that contribute to tumorigenesis (Zhang and Manley, 2013), we further compared the AS profile between SS and tumor-adjacent tissues by employing rMATS (Shen et al., 2014). Five classical splicing categories of exon skipping (ES), alternative 5′ donor sites (A5DS), alternative 3′ acceptor sites (A3AS), mutually exclusive exons (ME), and intron retention (IR) were analyzed. In total, we identified 2511 (including 41 lncRNA genes) significantly differential AS genes (DASGs), of which 2018, 223, 242, 486, and 159 belong to the splicing mode changes of ES, A5DS, A3AS, ME, and IR, respectively (Figure 2A, FDR <0.05, Supplementary Table S2). As expected, ES was the most common differential splicing mode (80.37%, 2018 out of 2511 DASGs), whereas IR was the least (6.33%, 159 out of 2511 DASGs). Notably, the majority of those DASGs among the five classical splicing categories were largely different, only a small portion of them simultaneously exhibited three or more distinct splicing types (Figure 2A).

Gene functional enrichment analysis indicated that those 2511 DASGs were mainly involved in the RNA splicing and cancer-related biological processes and KEGG pathways (Figures 2B,C, adjusted *p*-value < 0.05), which was highly correlated with the AS process. For instance, the top enriched biological processes of those DASGs were mRNA processing, microtubule cytoskeleton organization, and RNA splicing, while the enriched pathways are endocytosis, RNA transport, proteoglycans in cancer, and spliceosome. Moreover, we observed that 21 splicing factor genes showed significantly differential AS between SS and tumor-adjacent tissues, such as HNRNPA1, PTBP2, QKI, RBFOX2, and TRA2A. It is well known that the splicing factors are crucial for AS regulation (Lee and Rio, 2015), the deregulation of those splicing factors could drastically disrupt the splicing process of many corresponding genes and contribute to the tumorigenesis of SS (Dvinge et al., 2016). Furthermore, we found that 346, 204, and 122 oncogenes, TSGs, and TFs were also differentially spliced (Figure 2D). The abnormal splicing of these TFs could influence the expression of their downstream target genes and contribute to the development and progression of SS. Only 368 genes shared between DASGs and DEGs, leaving most of them were distinct (Figure 2E). These common 368 genes were enriched in the biological process of actomyosin structure organization and pathway of regulation of actin cytoskeleton (Figure 2E, adjusted *p*-value < 0.05). Thus, the genes that showed differential expression were quite distinct from those that exhibited differential splicing, suggesting that AS is complementary with expression level in revealing the transcriptomic changes. These results indicate that the abnormal AS changes of genes could be another important factor responsible for the tumorigenesis of SS.

### Dissection of the gene fusions in SS

**FIGURE 3:**
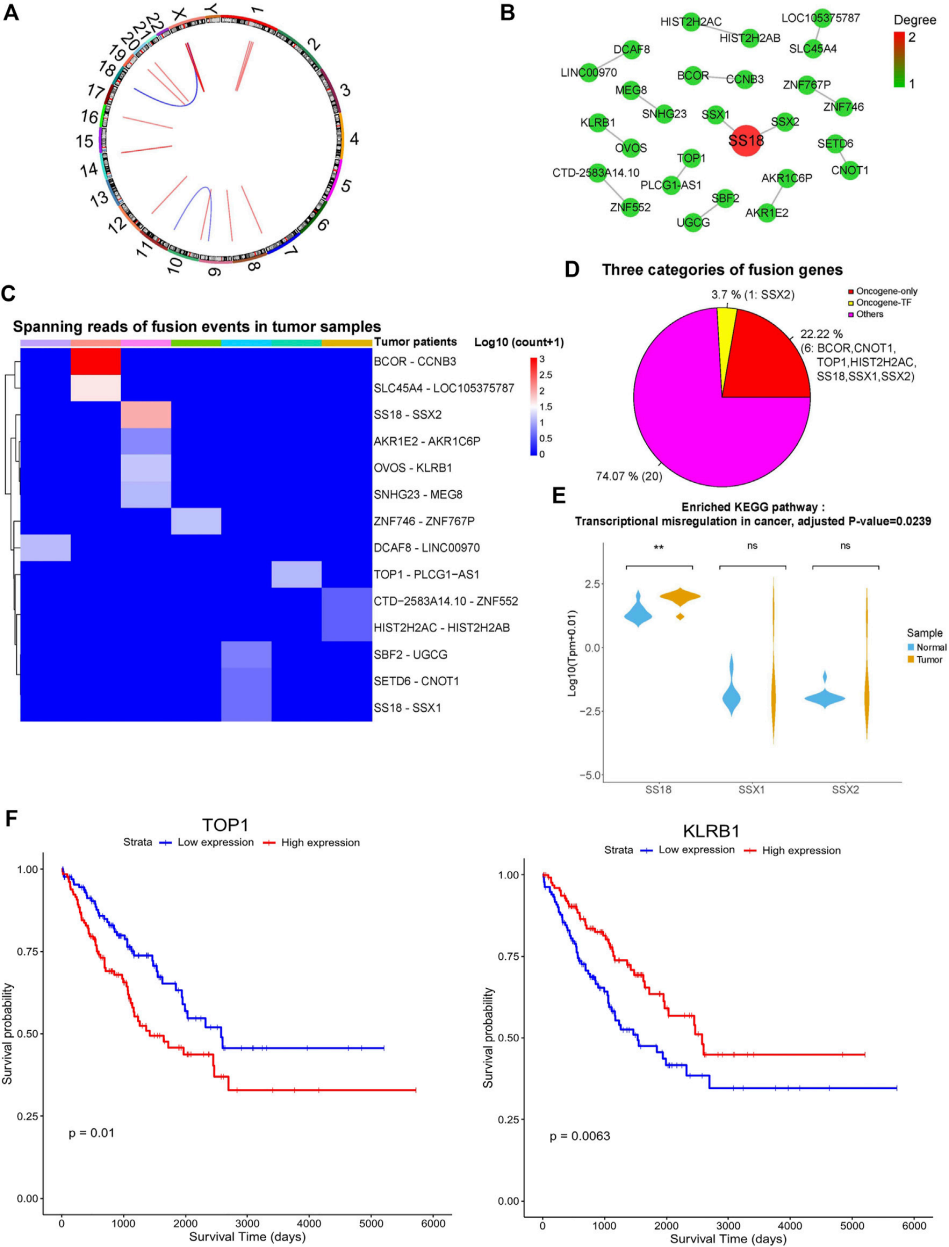
Gene fusion landscape of SS. **(A)** Circos plot showing the 14 tumor-specific fusion pairs in SS. **(B)** Network of tumor-specific fusion genes. The size and color of each circle correspond to the degree of fusion edges. **(C)** Heatmap displaying the supporting junction reads for tumor-specific gene fusions. **(D)** Different categories of the fusion genes. **(E)** Expression profile of the fusion genes involved in significantly enriched biological processes. Adjusted *p*-value < 0.05. **(F)** The fusion genes significantly correlated with the survival of TCGA sarcoma patients. *p*-value < 0.05.

We further explored the gene fusion events in SS patients using TopHat-Fusion (Kim and Salzberg, 2011). A total of 14 and 11 unique gene fusion pairs were separately identified in SS and tumor-adjacent tissues, and no fusion was shared between them. The 14 tumor-specific gene fusion pairs were from seven SS patients, most of which (11 out of 14) resulted from the rearrangements within the same chromosome, while 3 of them were generated by breaking and rejoining two disparate chromosomes (Figure 3A). In total, 27 genes were involved in these tumor-specific gene fusions. SS18 was fused with SSX1 and SSX2, which was in line with previous studies (Edwards, 2010). In contrast, other genes were mainly fused with one partner (Figure 3B).

As shown in Figure 3C, the maximum number of gene fusion pairs detected in individual patients was four and the gene fusion events in those SS patients were quite distinct. Intriguingly, these tumor-specific gene fusion events contain one TF of SSX2 and seven oncogenes of SS18, SSX1, SSX2, BCOR, CNOT1, HIST2H2AC, and TOP1 (Figure 3D). Oncogene SS18 was fused with the TF and oncogene of SSX2 as well as the oncogene SSX1, which is consistent with the known findings (Kawai et al., 1998). Besides, other oncogenes of BCOR, CNOT1, HIST2H2AC, and TOP1 formed the fusion events of BCOR-CCNB3, CNOT1-SETD6, HIST2H2AC-HIST2H2AB, and TOP1-PLCG1-AS1, respectively. Previous studies have shown that BCOR-CCNB3 fusion tends to occur in the undifferentiated small round-cell sarcomas like Ewing sarcoma and has the potential to drive sarcoma progression (Pierron et al., 2012; Li et al., 2016; Kao et al., 2018). Other gene fusions could be novel for SS, and the involved genes could be functionally important. For example, CNOT1 encodes the CCR4-NOT transcription complex subunit 1, which mainly participates in deadenylating mRNAs (Pavanello et al., 2018). HIST2H2AC and HIST2H2AB can generate the replication-dependent histones that are basic nuclear proteins responsible for the nucleosome structure of the chromosomal fiber. TOP1 encodes the enzyme of DNA topoisomerase for controlling and altering the topologic states of DNA during transcription (Baranello et al., 2016). Since TF could regulate the expression of many downstream target genes and oncogenes are closely associated with cancer, the fusion events of those TFs and oncogenes may contribute to the tumorigenesis/progression of SS. Interestingly, lncRNA genes of LINC00970, LOC105375787, and PLCG1-AS1 were also involved in the gene fusion events, but their functions were still unknown. Gene functional enrichment analysis showed that those fusion genes were significantly enriched in the KEGG pathway of transcriptional misregulation in cancer (Figure 3E, adjusted *p*-value < 0.05).

Moreover, we further explored the expression profile of these fusion genes using synovial sarcoma data from The Cancer Genome Atlas (TCGA) (Weinstein et al., 2013). As expected, these fusion genes showed similar expression patterns between the synovial sarcoma samples of us and TCGA (Supplementary Figure S3A). Additionally, we also found that these fusion genes exhibited slightly different expression profiles across distinct types of TCGA sarcomas (Supplementary Figure S3B). Considering that the number of synovial sarcoma samples is limited, we used all the TCGA sarcoma samples to do the survival analysis based on our identified fusion genes. Interestingly, the expression levels of KLRB1 and TOP1 were significantly associated with the survival of sarcoma patients (Figure 3F, *p* < 0.05), indicating that they could be potential prognostic markers.

### Circular RNAs may play a role in SS formation

**FIGURE 4:**
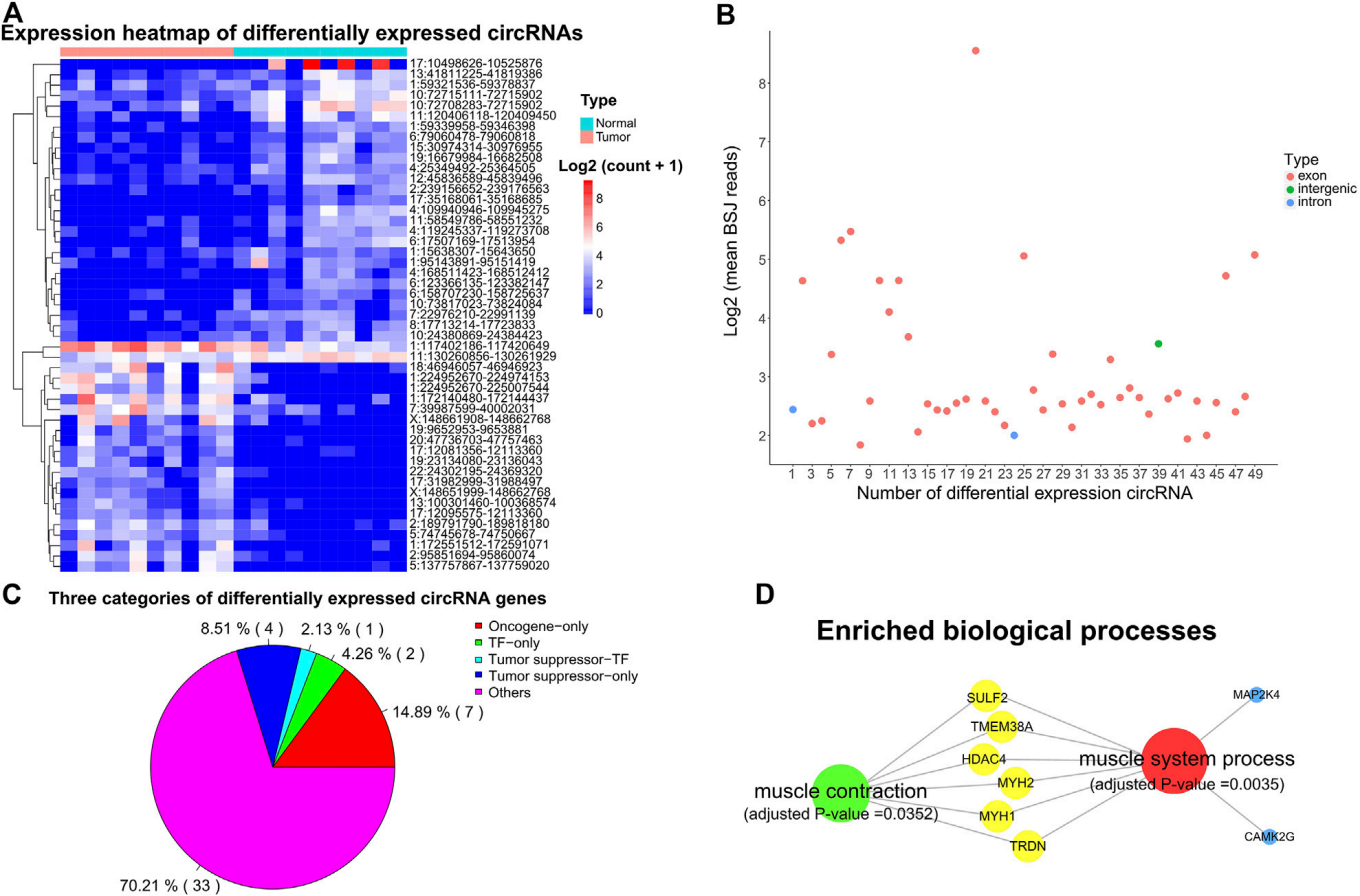
Expression profile and characteristics of circRNAs. **(A)** Expression heatmap showing the differentially expressed circRNAs. Supporting reads of circRNAs were used in the heatmap. **(B)** Genomic region of the differentially expressed circRNAs. BSJ: back-spliced junction. **(C)** Distinct types of the parental genes of differentially expressed circRNAs. **(D)** Significantly enriched biological processes for the parental genes of differentially expressed circRNAs. Adjusted *p*-value < 0.05.

Emerging evidence shows that circRNAs can involve in various aspects of tumor biology (Dong et al., 2017; Zhang and Xin, 2018), thus we further investigated the expression profile of circRNAs in SS and tumor-adjacent tissues. We detected 49 differentially expressed circular RNAs by employing CIRI with the threshold of |fold change| >2 and adjusted *p*-value < 0.01. As shown in Figure 4A, 21 of them were significantly up-regulated in SS, whereas the other 28 were down-regulated. Furthermore, we found that the great majority (46 out of 49, 93.88%) of those differentially expressed circRNAs were formed by the circulation of exons of their parental genes, only two circRNAs of 10:24380869|24384423 (parental gene: KIAA1217) and 17:35168061|35168685 (parental gene: UNC45B) were produced from the intronic region and another one (5:137757867|137759020) was generated in the intergenic region (Figure 4B).

Intriguingly, 7, 5, and 3 of the parental genes for those differentially expressed circRNAs were oncogenes, TSGs, and TFs (Figure 4C). Previous studies have shown that circRNAs can form posttranscriptional regulators to regulate the expression of their parental genes (Memczak et al., 2013; Zhang et al., 2013). Thus, these circRNAs have the potential to affect the expression of their parental oncogenes, TSGs, and TFs. Gene functional enrichment analysis showed that the parental genes of those differentially expressed circRNAs were mainly enriched in the muscle system process (such as MAP2K4, HDAC4, TMEM38A, MYH1, MYH2, CAMK2G, TRDN, and SULF2) and muscle contraction (e.g. HDAC4, TMEM38A, MYH1, MYH2, TRDN, and SULF2) (Figure 4D, adjusted *p*-value < 0.05).

### The genes involved in different types of transcriptomic changes are largely distinct

**FIGURE 5:**
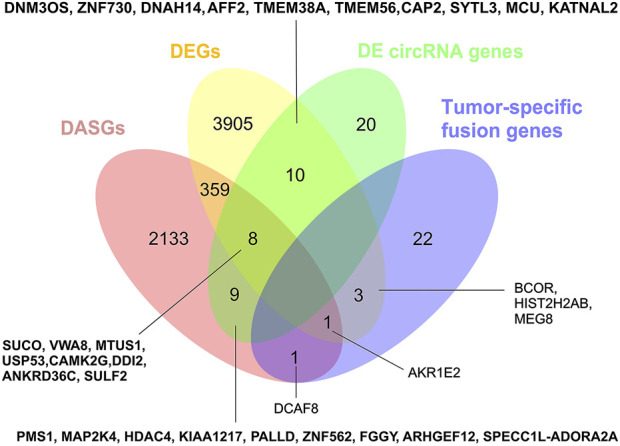
Comparison of DEGs, DASGs, fusion genes, and the parental genes of differentially expressed circRNAs. The overlapped genes for each part were shown in the figure. DEGs: differentially expressed genes; DASGs: differentially alternative spliced genes.

We further compared the four gene types of DEGs, DASGs, the fusion genes, and the parental genes of differentially expressed circRNAs. As shown in Figure 5, the genes in one type were largely distinct from that of other types, and no genes were common among the four categories. Only a fraction of them was involved in two or three types of changes (Figure 5). Intriguingly, the DEGs of BCOR, HIST2H2AB, and MEG8, and the DASGs of AKR1E2 and DCAF8 overlapped with the fusion genes, suggesting that the fusion events may influence the expression and/or AS profile of these genes. BCOR is an oncogene, while MEG8 is an imprinted gene. Moreover, 18 DEGs (e.g. DNM3OS, ZNF730, DNAH14, and AFF2) shared with the parental genes of differentially expressed circRNAs, implying that expression changes of these genes could affect the expression of circRNAs as well. In addition, 17 DASGs (such as SUCO, VWA8, MTUS1, and USP53) were common to the parental genes of differentially expressed circRNAs. Since circRNAs are mainly formed by AS of pre-mRNAs through backsplicing (Barrett and Salzman, 2016), the AS changes of these DASGs have the potential to influence the expression of corresponding circRNAs. Collectively, our results show that all the four transcriptomic aspects of expression changes, AS, gene fusions, and circRNAs could be closely correlated with the tumorigenesis/progression of SS.

### CircRNAs could potentially regulate the expression of a multitude of genes by acting as miRNA sponges

**FIGURE 6:**
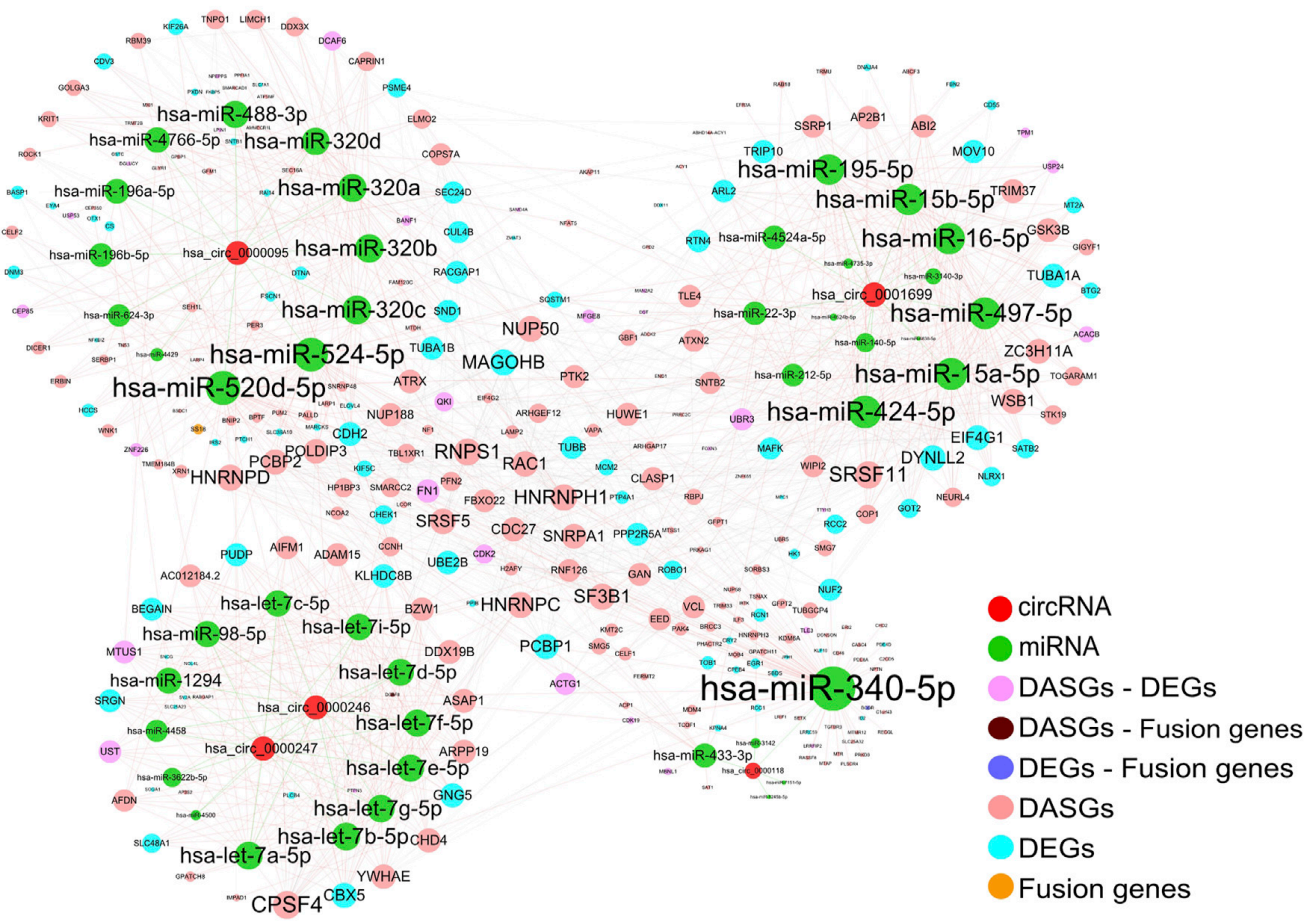
Interaction network among circRNAs, miRNAs, and related target genes. The edges represent the potential interactions between different types of genes and the size of each node is in proportion to the degree of edges. Only the target genes of DEGs, DASGs, fusion genes, and the parental genes of differentially expressed circRNAs were considered for corresponding miRNAs. The protein-protein interactions were obtained from the STRING database, while the known circRNA -miRNA interactions were downloaded from starBase. The resulting interaction network contains 5 circRNAs, 44 miRNAs, and 293 protein-coding genes. DEGs: differentially expressed genes; DASGs: differentially alternative spliced genes.

An increasing number of studies suggested that endogenous circRNAs can act as miRNA sponges to regulate corresponding gene expression (Kulcheski et al., 2016; Panda, 2018). We further constructed the interaction network among differentially expressed circRNAs, miRNAs, and the miRNA target genes of DEGs, DASGs, and fusion genes to elucidate the functional roles of those differentially expressed circRNAs. Based on the known miRNA-circRNA regulations, and the miRNA-targets relationships in the starBase database (Li et al., 2014) as well as the protein-protein interactions (PPIs) in the String database (Szklarczyk et al., 2019), the resulting interaction network involved in 5 circRNAs (hsa_circ_0001699, hsa_circ_0000247, hsa_circ_0000246, hsa_circ_0000095, and hsa_circ_0000118), 44 miRNAs, 293 protein-coding genes, containing 57 miRNA-circRNA interactions, 789 miRNA-mRNA interactions and 350 PPIs (Figure 6).

It is well known that circRNAs can regulate gene expression by influencing transcription, mRNA turnover, and translation by sponging RNA-binding proteins (RBPs) and miRNAs (Panda, 2018). Our resulting network showed that circRNAs hsa_circ_0001699, hsa_circ_0000247, hsa_circ_0000246, hsa_circ_0000095, and hsa_circ_0000118 could act as the sponges of 14, 13, 13, 12, and 5 miRNAs, respectively. Moreover, these miRNAs have the potential to regulate the expression of 119, 202, and 3 genes of DEGs, DASGs, and/or fusion genes. Based on the findings in previous studies (Kulcheski et al., 2016; Panda, 2018). The expression of these miRNA target genes could be indirectly influenced by corresponding circRNAs through competing for the interaction with miRNAs. Consequently, our result suggests that circRNAs could potentially function as miRNA sponges to regulate the expression of an abundance of corresponding genes.

## Discussion

In this study, we systematically explored the transcriptome alterations of SS in terms of gene expression and AS, as well as gene fusions and circRNAs. A total of 4286 genes (including 765 lncRNA genes) were differentially expressed between SS and paired tumor-adjacent tissues, which were mainly involved in fundamental biological processes and cancer-related pathways. Moreover, we experimentally validated the differential expression of seven splicing factors using qPCR. We also detected 2511 genes (including 41 lncRNA genes) that showed differential AS, where the most common AS mode was ES (80.37% of these DASGs), followed by ME, A3AS, A5DS, and IR. Gene functional enrichment analysis also showed that these DASGs were enriched in splicing-related biological processes and pathways. Surprisingly, those DEGs and DASGs were largely distinct, only a small portion of them were the same, suggesting that AS is complementary with expression level for investigating transcriptomic changes. Notably, a fraction of those DEGs and DAGs were oncogenes, tumor suppressors, and TFs, indicating that they could be closely associated with the tumorigenesis of SS. Moreover, we identified 14 tumor-specific gene fusion pairs in SS, which not only included the known gene fusions of SS18-SSX but also contained novel fusion events involving both protein-coding and lncRNA genes. Additionally, we observed that 49 circRNAs markedly changed expression in SS compared to tumor-adjacent tissues, and their parental genes were enriched in the muscle system process.

To the best of our knowledge, we are the first to study the SS transcriptome from a comprehensive view covering both transcriptional and post-transcriptional levels. Specifically, the deregulation of AS and the role of circRNAs were rarely explored in SS previously. An increasing number of studies have shown that imbalances in the AS process can affect the development of various human diseases, especially the oncogenesis, progression, and metastasis of a range of cancers (Scotti and Swanson, 2016). We identified 122 differentially spliced TFs and 124 differentially expressed TFs, suggesting that these TFs could be responsible for the expression level changes of an abundance of their target genes (Vaquerizas et al., 2009; Lambert et al., 2018). Moreover, we observed that 7 and 21 splicing factors were dramatically changed in expression level or AS profile. Since splicing factors are essential in regulating the AS of genes, these abnormally changed splicing factors may significantly contribute to the AS changes of many related genes (Anczukow and Krainer, 2016). On the other hand, circRNAs have critical regulatory functions and play key roles in the initiation and progression of diverse diseases including cancers (Zhang et al., 2018; Haddad and Lorenzen, 2019). The differentially expressed circRNAs identified by us were mainly generated from the genes correlated with the muscle system process and contraction. We also constructed the interaction network among circRNAs, miRNAs, and downstream target genes to elucidate their potential regulatory mechanism. The resulting network indicated that those differentially expressed circRNAs have the potential to act as the sponge for dozens of miRNAs to indirectly regulate the expression of hundreds of DEGs and DASGs.

## Conclusion

Collectively, we systematically dissected the transcriptomic profile of SS and identified a number of DEGs, DASGs, fusion genes, and circRNAs that could be closely associated with the tumorigenesis of SS. Our study not only gained novel insights into SS transcription and post-transcription but also shed light on the underlying molecular mechanisms.

## Data Availability

The data presented in the study are deposited in the Gene Expression Omnibus (GEO) repository, https://www.ncbi.nlm.nih.gov/geo/query/acc.cgi?acc=GSE144190.190, accession number GSE144.
