# Supplementary material for: Integrative analysis of synovial sarcoma transcriptome reveals different types of transcriptomic changes
Source: Front Genet. 2022 Sep 2;13:925564. doi: 10.3389/fgene.2022.925564 (PMC9478865; doi:10.3389/fgene.2022.925564)
Supplement: Supplementary file 1 [file Table1.DOCX]

**Table 1.** Detailed information of 10 synovial sarcoma patients.

| **Patient ID** | **Age** | **Sex** | **Tumor Location** | **Tumor Size (cm)** | **Tumor status** | **Outcome** |
| --- | --- | --- | --- | --- | --- | --- |
| 1 | 26 | Female | Thigh | 12*10.5*10 | Primary | Alive |
| 2 | 18 | Male | Foot | 6.0*3.5*2.0 | Local recurrence | Died |
| 3 | 37 | Male | Groin | 9.5*6*6 | Local recurrence | Alive |
| 4 | 29 | Female | Lung | 2.0*2.0*1.3 | Primary | Alive |
| 5 | 59 | Female | [Iliac Bone](javascript:;) | 5.5*4.5*3.5 | Local recurrence | Alive |
| 6 | 28 | Female | Foot | 2*1.7*0.7 | Primary | Alive |
| 7 | 20 | Female | Neck | 6*5*4 | Primary | Alive |
| 8 | 27 | Male | [Shank](javascript:;) | 7*6*2 | Primary | Alive |
| 9 | 41 | Male | [Shank](javascript:;) | 8*6*4 | Local recurrence | Alive |
| 10 | 71 | Female | Thigh | 9*6*3 | Primary | Alive |
